# Digital Inclusion Pathways for Older Chinese Adults in the Context of Active Aging: Secondary Analysis of 2023 China Longitudinal Aging Social Survey Data

**DOI:** 10.2196/83078

**Published:** 2026-03-17

**Authors:** Bo Zhao, Xiaoyan Wang, Ren Chen, Eun Woo Nam

**Affiliations:** 1 Department of Health Services Management, School of Health Services Management, Anhui Medical University, 81 Meishan Road, Shushan District, Hefei, Anhui, China, 86-18550072738; 2 Yonsei Global Health Center, Yonsei University, 1 Yonseidae-gil, Wonju-si, Gangwo-do, Republic of Korea

**Keywords:** digital inclusion, social engagement, mental health, overall health status, healthy aging, active aging, older adults, China

## Abstract

**Background:**

Rapid population aging and the intensifying digitalization of everyday life are unfolding simultaneously in China. While prior studies have largely examined pairwise associations among digital inclusion, social engagement, mental health, and overall health status, few have evaluated an integrated, theoretically grounded pathway linking these domains in later life.

**Objective:**

This study aims to quantify the direct and indirect pathways through which digital inclusion influences older adults’ overall health status, social engagement, and mental health, specified as sequential mediators.

**Methods:**

We analyzed the newly released, nationally representative data from the 2023 wave of the China Longitudinal Aging Social Survey, comprising 9918 adults aged 60 years or older. Overall health status was assessed using 3 self-rated health (SRH) indicators: current SRH, SRH relative to age peers, and SRH relative to last year. Digital inclusion was measured through digital access, device proficiency, and digital ability. Social engagement captured social support, frequency of participation in community or voluntary activities, and nononline activities. Mental health included depressive symptoms, social adaptation, and life satisfaction. Analyses included descriptive statistics, multivariable hierarchical linear regressions, and structural equation modeling to estimate direct and mediated effects (2-sided*;* α=.05).

**Results:**

Older age, chronic disease, and functional limitations were associated with poorer overall health status, whereas higher education and current employment were associated with better health status. Digital inclusion was positively associated with social engagement (β=.50), which in turn was positively associated with mental health. Mental health showed the strongest association with SRH (β=.74). The direct path from social engagement to overall health status was nonsignificant (*P*=.34), indicating that participation influences health primarily through psychological pathways. In regression analyses, digital inclusion modestly improved model fit for health status outcomes, while adding mental health produced a greater increase.

**Conclusions:**

Digital inclusion promoted active aging indirectly, by expanding social engagement and enhancing mental health, thereby improving overall health status. Policy efforts should prioritize narrowing the digital divide by improving digital skills and capability, rather than access alone. Meaningful opportunities for social engagement should also be expanded to strengthen community-based mental health support. In addition, strategies should be tailored to the differing needs of urban and rural settings.

## Introduction

### Background

Population aging has become one of the most consequential demographic shifts worldwide. In China, this transition is particularly rapid: by the end of 2024, adults aged 60 years or older accounted for 22% of the national population [1], accompanied by rising multimorbidity, functional decline, and widening social and health disparities. As chronic disease burden and long-term care needs increase, understanding the mechanisms that support health and well-being in later life has become a central scientific and policy priority [2].

To guide global responses, the Active Ageing Framework of the World Health Organization (WHO) emphasized that healthy aging is not only about disease prevention but also about creating an environment that enables older adults to remain healthy and socially engaged [3]. This perspective aligns with core gerontological theories: the activity theory highlights the importance of meaningful participation for psychological and social well-being [4], while the socioemotional selectivity theory underscores the increasing value of emotionally meaningful social ties in later life [5]. China has integrated these principles into national strategies, illustrated by the 2025 *Guiding Opinions on Supporting Elderly People’s Social Participation and Promoting the Realization of a Proactive Age*, which call for the need to mobilize older adults’ capacities, enhance opportunities for social engagement and overall health, and reduce psychosocial risks [6].

Meanwhile, the rapid digitalization of everyday life has profoundly reshaped how older adults access services, maintain social ties, and engage with community resources. By 2024, China had 157 million internet users aged 60 years or older, yet their online participation remains disproportionately low [7,8]. Digital inclusion today extends beyond access to encompass skills, motivation, trust, and the ability to meaningfully participate in digital environments. The digital divide theory conceptualizes these disparities as layered barriers—spanning access, ability, and confidence—that structure opportunities for social interaction and integration [9,10]. For many older adults, insufficient digital competence, low usability of platforms, and the absence of age-friendly design restrict meaningful digital participation and may exacerbate social and health inequalities.

An integrated theoretical perspective suggests that digital inclusion can influence well-being through psychosocial pathways [11]. The removal of access and skill barriers expands older adults’ capacity to obtain information and mobilize social support, forming the structural basis for the participation mechanisms described by the activity theory. Technology acceptance frameworks explain how perceived usefulness, ease of use, and digital self-efficacy determine whether digital access translates into sustained digital engagement [12]. The biopsychosocial model further posits that psychological well-being—shaped in part by social engagement—is a central determinant of both perceived and actual health [13]. Together, these perspectives imply a sequential logic in which digital inclusion facilitates social engagement, which enhances mental health, ultimately contributing to better overall health [14].

However, most existing studies examine these components in isolation—for example, exploring associations between digital use and social connectedness, or between social engagement and mental health—without testing how these elements interact as part of a continuous mechanism [11]. The absence of an integrated analytic framework limits both theoretical coherence and the development of targeted interventions to promote active aging within increasingly digital environments. Empirical validation of the full pathway linking digital inclusion, social engagement, mental health, and their overall health is therefore essential for understanding how digitalization intersects with aging and for informing policy efforts that support healthy, meaningful, and socially integrated later life [15].

### Literature Review

Existing research provides substantial but fragmented evidence on the individual links within the broader connecting digital inclusion to health in later life. Studies commonly assess digital inclusion across access, skills, use frequency, and trust and report significant positive effects on social interaction and meaningful forms of participation [16-18]. Digital engagement extends beyond leisure use to include health information seeking, online consultations, communication with family, and participation in community activities [19]. These digitally enabled interactions have been shown to reduce social isolation, strengthen support networks, and enhance older adults’ ability to mobilize social resources. Subgroup analyses further indicate heterogeneous effects, with women, urban residents, and younger segments of the older adult population benefiting more from digital inclusion [20]. While literature consistently affirms the positive association between digital engagement and social participation, most studies examine direct associations and rarely evaluate how digital inclusion may activate deeper psychosocial processes that ultimately shape well-being.

Research on the linkage between social engagement and mental health provides equally strong evidence yet remains similarly limited. Social engagement is widely recognized as a cornerstone to active aging. Numerous empirical studies demonstrate that greater frequency and diversity of participation are associated with lower depressive symptoms, reduced loneliness, improved social adaptation, and higher life satisfaction [21-25]. Engagement enhances psychological resources by offering meaningful roles, reinforcing identity and purpose, and strengthening emotional connections—all of which align with core propositions of the biopsychosocial model. Mental health, in turn, as the central determinant of overall health and self-rated health (SRH), exerts substantial influence through mechanisms such as stress buffering, resilience, and positive cognitive appraisal [21-27]. Although these findings underscore mental health as a pivotal conduit between social participation and health outcomes, prior studies rarely embed mental health within a continuous pathway that begins with digital inclusion and culminates in overall health [17,28]. Consequently, the literature lacks an integrated explanation of how digitalization interacts with social processes and psychological well-being to shape health trajectories in later life.

To address these gaps, this study draws on the 2023 wave of the China Longitudinal Aging Social Survey (CLASS), the most recently released and nationally representative dataset on Chinese older adults. Importantly, the data were collected in the immediate post–COVID-19 period, during which digital technologies became deeply embedded in daily life and the digital divide among older adults intensified. This context offered a unique opportunity to examine the mechanisms through which digital inclusion influences social engagement, mental health, and overall health. By constructing and testing an integrated pathway model, this study offers timely empirical evidence to inform more precise and actionable strategies for promoting digital inclusion and active aging in China’s rapidly aging society.

## Methods

### Study Design and Participants

This study used a cross-sectional design and analyzed data from the CLASS 2023. The CLASS project is a national, large-scale social survey conducted by the National Survey Research Center at Renmin University of China. The survey covered 28 provinces, 134 districts, and 462 villages across China, excluding Hong Kong, Taiwan, Macao, Hainan, Xinjiang, and Tibet. The survey collected detailed information on respondents’ health status, family structure, social background, and economic conditions.

### Ethical Considerations

The study of the CLASS database was approved by the ethics committee at the Capital University of Physical Education and Sport (ChiCTR-IOR-ChiCTR2200063177). All participants provided informed consent before, and for individuals with limited literacy skills, consent was obtained from their legally authorized representatives. Participants were informed of the study purpose and their right to withdraw at any time. All data were deidentified, stored securely, and accessible only to authorized research team members to ensure privacy and confidentiality. No financial compensation was provided, and the study involved minimal risk.

### Survey Procedures

Data collection for the CLASS project was carried out using the China Social Survey Network. The survey used a stratified, multistage probability sampling method. County-level regions, including counties, county-level cities, and districts, were selected as primary sampling units. Villages and neighborhood committees served as secondary sampling units. With each secondary sampling unit, sample households were randomly selected, and 1 eligible older adult was interviewed per household. Data were collected through structured interviews and standardized questionnaires. To ensure data quality, on-site supervision and call-backs were employed as part of the quality control measures implemented by CLASS.

### Variables

The dependent variable was older adults’ overall health status, proxied by SRH using 3 items: current SRH (respondents rate their present health status, scores of 0‐5), SRH relative to age peers (scores of 0‐5), and SRH relative to last year (scores of 0‐3). Item scores were summed (range 3‐13), with higher scores indicating better overall health.

Independent variables included measures of digital inclusion and active aging indicators. Digital inclusion comprised 3 dimensions as follows:

- Digital access: This dimension reported whether the respondent could connect to the internet via available infrastructure and devices, measured by three items—network availability at the dwelling; current internet use; and ability to access the internet via a mobile phone, tablet, or computer. Summed scores reflected overall access, with higher scores indicating greater access.
- Device proficiency: This dimension reported self-rated proficiency in operating devices such as mobile phones and tablets, rated from 1 (“very unskilled”) to 5 (“very skilled”). Higher summed scores indicated stronger digital skills.
- Digital ability: This dimension reported the performance of 11 common online activities (eg, voice or video chat, SMS text messaging, online shopping, and reading news), each scored from 0 to 1. Total scores (range 0‐11) reflected functional digital competence.

Active aging comprises health, engagement, and security; because security is primarily a macro-level construct [29], this study focused on individual-level with health and engagement. Social engagement included 3 components as follows:

- Social support and network: This component was a six-item scale measuring (1) the number of family or relatives contacted or met at least monthly, (2) the number of family or relatives with whom one can confidently discuss private matters, (3) the number of family or relatives available to help when needed, and (4-6) the same three items for friends. Each item was scored from 0 to 5; total scores ranged from 0 to 30, with higher scores indicating stronger support and denser network.
- Activity participation frequency: This component measured involvement in 7 community or voluntary activities (community patrols, caring for older adults or children, environmental protection, dispute mediation, companion chatting, professional volunteering, and caring for the next generation). Each item was scored from 0 to 4 (0=“never” to 4=“almost daily”); total scores ranged from 0 to 28.
- Nononline activities: This component measured participation in 6 offline activities (religious activities; senior college or training courses; watching television, listening to the radio, reading newspapers or books, or watching an opera; singing or playing instruments; mahjong, chess, or cards; and square dancing). Each item was scored from 0 to 4; total scores ranged from 0 to 24, with higher scores indicating greater offline engagement.

Mental health included 3 measures as follows:

- Depressive symptoms: This component was measured using the 9-item short form of the Center for Epidemiologic Studies Depression Scale [30]. Items were scored from 1 to 3 (1=“no,” 2=“sometimes,” and 3=“often”). Except for items 1, 4, and 9, all items were reverse-scored, producing a total score that ranged from 9 to 27, with higher scores indicating fewer depressive symptoms.
- Social adaptation: This component was measured using 8 items (eg, willingness to participate in village or neighborhood committee work, desire to contribute to society again, current enjoyment of learning, feeling useful to society, difficulty adapting to rapid social change, difficulty accepting more viewpoints, difficulty accepting new social policies, and perception that social change is increasingly unfavorable to older adults). Items were scored from 1 to 5 (1=“strongly disagree” to 5=“strongly agree”); total scores range from 8 to 40, with higher scores indicating better adaptation.
- Life satisfaction: This was a single global item measuring overall life satisfaction (1=“very dissatisfied” to 5=“very satisfied”).

Following prior studies [18,31], this study included a set of covariates to control for demographic and health-related factors, including age, sex, educational level, marital status, number of coresidents, household registration (*hukou*), chronic disease (yes or no), and current employment status (yes or no). Functional status measures included as follows:

1. Activities of daily living (ADL): 11 items scored from 1 to 3 (1=“independent,” 2=“needs some help,” and 3=“unable”); total scores ranged from 11 to 33.
2. Instrumental activities of daily living: 9 items scored from 1 to 3 (same as ADL); total scores range from 9 to 27, where lower scores indicated better instrumental functioning.

### Statistical Analysis

#### Power

Data were curated in SPSS version 26.0 (IBM Corp). We conducted descriptive analyses, chi-square tests, correlation analyses, and multivariable hierarchical regression. Structural equation modeling (SEM) was specified in AMOS version 28.0 (IBM Corp); model fit was evaluated and modified as appropriate. It also compared pathways by which digital inclusion, social engagement, and mental health influence older adults’ overall health status. Statistical significance was set at a 2-sided α of .05.

#### Data Exclusion

The 2023 CLASS dataset contains 11,670 valid cases. Based on the requirements of the research design, variables unrelated to the study aims were excluded, and missing data were processed accordingly. A final analytic sample of 9918 respondents was obtained.

## Results

### Descriptive Analysis

**Figure 1.**
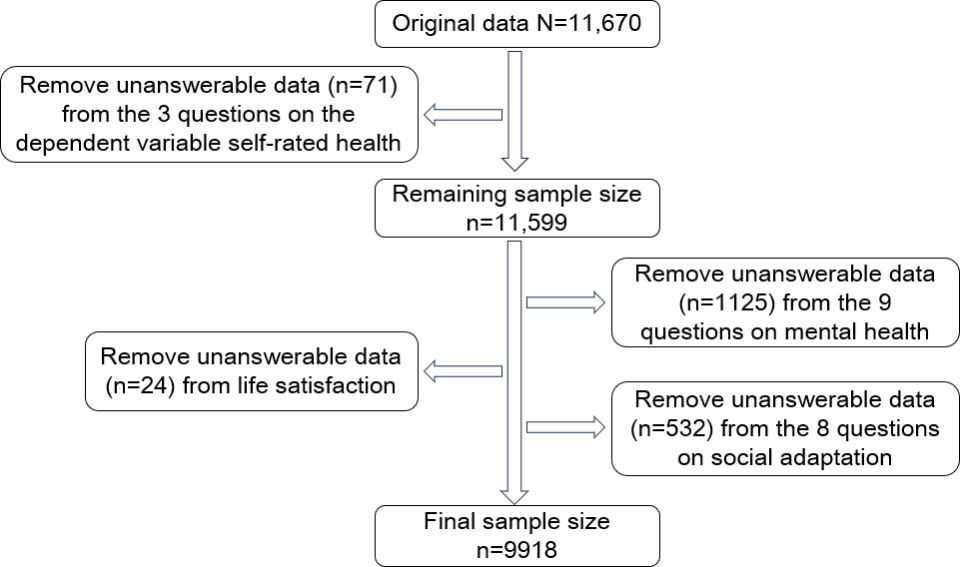
Data exclusion flowchart.

Table 1 showed significant differences in SRH across all demographic groups (*P*<.001). Younger respondents and those with higher education, smaller household sizes, nonrural *hukou*, absence of chronic diseases, and current employment all reported higher SRH. Men also showed slightly better SRH than women. Overall, health status was consistently better among individuals with greater socioeconomic resources and fewer health limitations (Figure 1).

**Table 1.** Baseline sociodemographic characteristics (n=9918).

Variable	Value, n (%)	SRH^a^, mean (SD)	2-tailed *t*/*F* test (*df*)	*P* value
Sex			4.938^b^ (9916)	<.001
Male	5121 (51.6)	8.81 (1.507)		
Female	4797 (48.4)	8.66 (1.519)		
Age (y)			15.395^b^ (9916)	<.001
60-74	7491 (75.5)	8.87 (1.503)		
≥75	2427 (24.5)	8.33 (1.476)		
Marital status			–8.056^b^ (9916)	<.001
Married or with spouse	8209 (82.8)	8.80 (1.490)		
Other	1709 (17.2)	8.46 (1.596)		
Education			88.915^c^ (2, 9915)	<.001
Illiterate	1709 (17.2)	8.46 (1.596)		
Junior middle school or below	6791 (68.5)	8.72 (1.495)		
High school or above	1418 (14.3)	9.17 (1.406)		
Coresidents			13.404^b^ (9916)	<.001
1–2 persons	7262 (73.2)	8.87 (1.460)		
≥3 persons	2656 (26.8)	8.39 (1.603)		
Household registration (*hukou*)			–3.613^b^ (9916)	<.001
Rural	5183 (52.3)	8.69 (1.557)		
Nonrural	4735 (47.7)	8.80 (1.464)		
Chronic disease			28.674^b^ (9916)	<.001
No	2047 (20.6)	9.56 (1.415)		
Yes	7871 (79.4)	8.53 (1.465)		
Current employment			−9.143^b^ (9916)	<.001
Not working	7069 (71.3)	8.65 (1.529)		
Working	2849 (28.7)	8.95 (1.455)		

a SRH: Self-rated health.

b *t* test.

c *F* test.

Table 2 presented descriptive statistics for the main study variables. Mean ADL and instrumental activities of daily living scores were 11.38 and 9.58, respectively. SRH measures (3.59, 3.23, and 1.92) showed moderate levels of perceived current and comparative health. Digital inclusion indicators (access, proficiency, and ability) displayed relatively low mean scores with wide variability. Social engagement levels were modest overall, while social support was relatively higher. Mental health indicators showed moderate depressive symptoms, average social adaptation, and relatively high life satisfaction.

**Table 2.** Descriptive statistics of key variables.

Domain	Score, minimum	Score, maximum	Score, mean (SD)	
Functional status				
ADL^a^	11	33	11.38 (1.515)	
IADL^b^	9	25	9.58 (1.802)	
Overall health status (SRH^c^)				
Current SRH (1-5)	1	5	3.59 (0.78)	
SRH vs age peers (1-5)	1	5	3.23 (0.666)	
SRH vs last year (1-3)	1	3	1.92 (0.391)	
Digital inclusion				
Digital access (0-5)	0	5	1.77 (1.395)	
Device proficiency (0-15)	0	15	3.84 (3.983)	
Digital ability (0-11)	0	11	2.22 (2.627)	
Social engagement				
Activity participation frequency (0-28)	0	25	1.86 (3.862)	
Nononline activities (0-24)	0	23	5.68 (3.555)	
Social support (0-30)	0	30	14.39 (4.955)	
Mental health				
Depressive symptoms (CES-D^d^ short, 9-27)^e^	9	27	15.48 (3.386)	
Social adaptation (8-40)	8	40	24.53 (4.064)	
Life satisfaction (1-5)	1	5	3.84 (0.733)	

a ADL: activities of daily living.

b IADL: instrumental activities of daily living.

c SRH: self-rated health.

d CES-D: Center for Epidemiologic Studies Depression Scale.

e Higher scores indicate fewer depressive symptoms (lower depressive tendency).

### Results of Regression Analysis

Table 3 presented the hierarchical regression results for overall health. Model 1, which included demographic and baseline health variables, explained 16.4% of the variance in overall health (Δ*R*²=0.164). Older age, being unmarried, larger household size, poorer functional status, and having chronic conditions were all negatively associated with health, whereas higher education and being employed showed positive associations. Model 2 added digital inclusion variables and modestly increased explanatory power (Δ*R*²=0.173). Digital proficiency and digital ability—rather than mere access—were positively associated with better perceived health. Model 3 incorporated social engagement indicators but did not meaningfully improve model fit (Δ*R*² unchanged). Model 4 added mental health variables and substantially increased explanatory power to 29.4% (Δ*R*²=0.294). Lower depressive symptoms, higher life satisfaction, and better social adaptation emerged as strong predictors of overall health.

**Table 3.** Hierarchical regression for the overall health status (self-rated health).

Variables	Model 1		Model 2		Model 3		Model 4	
	Value	*P* value	Value	*P* value	Value	*P* value	Value	*P* value
Constant	13.083	<.001	12.324	<.001	12.327	<.001	8.178	<.001
Age	−.017	<.001	−.007	.01	−.007	.01	−.010	<.001
Education	.326	<.001	.209	<.001	.209	<.001	.104	.01
Marital status	−.198	.001	−.155	.01	−.155	.01	−0.085	.13
Coresidents	−.209	<.001	−.195	<.001	−.191	<.001	−.159	<.001
ADL^a^	−.033	.03	−.041	.006	−.040	.008	−.028	.04
IADL^b^	−.162	<.001	−.157	<.001	−.157	<.001	−.139	<.001
Chronic disease	−.835	<.001	−.851	<.001	−.850	<.001	−.585	<.001
Current Employment	.228	<.001	.267	<.001	.274	<.001	.277	<.001
Digital inclusion								
Digital access	—^b^	—	0.014	.54	0.019	.43	−0.009	.70
Device proficiency	—	—	.018	.03	0.017	.06	0.012	.15
Digital ability	—	—	.036	<.001	.032	.001	0.002	.79
Social engagement								
Activity frequency	—	—	—	—	0	.92	0.003	.35
Nononline activities	—	—	—	—	0.007	.13	−0.003	.51
Social support	—	—	—	—	−0.005	.09	−.009	.001
Mental health								
Depressive symptoms	—	—	—	—	—	—	.053	<.001
Life satisfaction	—	—	—	—	—	—	.636	<.001
Social adaptation	—	—	—	—	—	—	.014	<.001
*F* test (*df*)	243.780 (8, 9909)	<.001	190.043 (11, 9906)	<.001	149.679 (14, 9903)	<.001	244.467 (17, 9900)	<.001
*R*²	0.164	—	0.174	—	0.175	—	0.296	—
△*R*²	0.164	—	0.173	—	0.173	—	0.294	—

a ADL: activities of daily living.

b Data not available.

To verify the robustness of the baseline regression results, a sensitivity analysis was also conducted. As some areas may introduce bias to the estimated digital inclusion effects, respondents from 3 regions with the highest levels of internet development during the survey period (Guangdong, Shanghai, and Beijing) were excluded. Using the remaining sample, we re-estimated the pathway linking digital inclusion, social engagement, mental health, and overall health. The results of the revised model were highly consistent with models in Table 3, indicating strong robustness of the estimated relationships (Table 4).

**Table 4.** Results of the robustness analysis.

Variables	Model 5		Model 6		Model 7		Model 8	
	Value	*P* value	Value	*P* value	Value	*P* value	Value	*P* value
Digital inclusion								
Digital access	—^a^	—	−0.011	.68	−0.009	.73	−0.04	.10
Device proficiency	—	—	0.016	.10	0.013	.21	0.011	.23
Digital ability	—	—	.064	<.001	.061	<.001	.032	.002
Social engagement								
Activity frequency	—	—	—	—	−0.002	.66	−0.001	.81
Nononline activities	—	—	—	—	.014	.005	0.003	.52
Social support	—	—	—	—	−0.001	.76	−.006	.04
Mental health								
Depressive symptoms	—	—	—	—	—	—	.055	<.001
Life satisfaction	—	—	—	—	—	—	.617	<.001
Social adaptation	—	—	—	—	—	—	.019	<.001
Sample sizes	8533	—	8533	—	8533	—	8533	—
Control variables	Yes	—	Yes	—	Yes	—	Yes	—
*F* test (*df*)	209.194 (8, 8524)	<.001	167.196 (11, 8521)	<.001	132.007 (14, 8518)	<.001	211.313 (17, 8515)	<.001
*R*²	0.164	—	0.178	—	0.178	—	0.297	—
△*R*²	0.163	—	0.176	—	0.177	—	0.295	—

### SEM Analysis Outcomes

**Figure 2.**
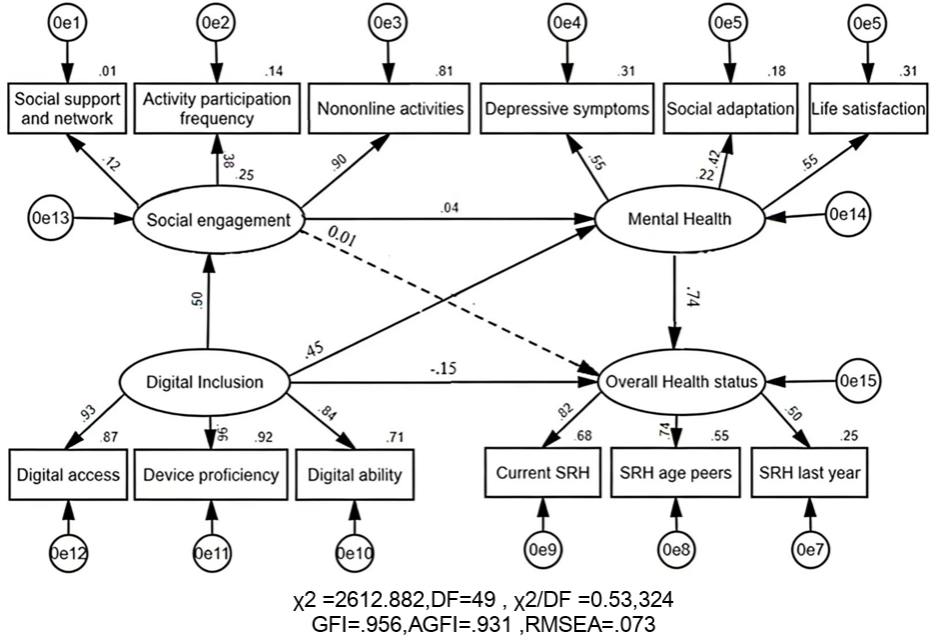
Structural equation model linking digital inclusion, social engagement, mental health, and overall health status. DF: degree of freedom; GFI: goodness-of-fit index; AGFI: adjusted goodness-of-fit index; RMSEA: root mean square error of approximation; SRH: self-rated health.

Figure 2 showed that the structural equation model identified multiple significant pathways linking digital inclusion, social engagement, mental health, and overall health status. Digital inclusion strongly predicted social engagement (β=.45) and influenced overall health indirectly through its effects on engagement and, subsequently, mental health. Although the direct path from social engagement to overall health was negligible (β=.01), its effect on mental health was significant (β=.04). Mental health had the strongest predictive power in the model (β=.74). Surprisingly, digital inclusion had a small negative direct effect on overall health (β=–0.15), although the indirect pathways through engagement and mental health were positive. Model fit indices indicated acceptable fit (goodness-of-fit index=0.956, adjusted goodness-of-fit index=0.931, and root mean square error of approximation=0.073).

## Discussion

### Principal Results

This study elucidated how demographic characteristics, digital inclusion, and social engagement and mental health jointly shape older adults’ perceptions of overall health. First, results showed that advancing age, chronic disease burden, and functional limitations were associated with poorer SRH, consistent with longitudinal evidence from multiple countries. Specifically, SRH declines with age—likely reflecting physiological deterioration, cumulative adverse life events, and shifting social roles—and age is a robust predictor of SRH among older adults [32]. Chronic diseases also weakened perceived overall health, reinforcing findings that multimorbidity served as a risk factor for poor SRH [33]. Internationally, analyses using the Health and Retirement Study (HRS) and the English Longitudinal Study of Ageing (ELSA) showed that a higher number of conditions was tightly linked to lower SRH, even after psychological factors were considered [34]. Functional limitations further depressed health perceptions from a capability standpoint. By contrast, higher educational attainment and current employment operated as stable protective factors, suggesting that education^22^ and work [22] not only improved health literacy and economic resources but also expanded social support networks that buffer the health costs of aging. These associations aligned with cumulative advantage theory and underscore how late-life health inequalities reflect long-term disparities in socioeconomic and social resource accumulation.

Second, the findings clarified the mechanism of digital inclusion as one of “limited direct effects but substantial indirect effects.” Despite high factor loadings for digital access, device proficiency, and digital ability—and a clear positive effect of digital inclusion on social engagement—digital inclusion did not directly translate into better overall health. This mirrors evidence that where uptake of digital health services (eg, teleconsultation and health management apps) remains modest, digital skills training without integrated health education or health intervention yields limited direct health gains. Survey of Health, Ageing and Retirement in Europe (SHARE) data indicated that internet use had only a weak direct association with SRH. Its benefits are realized mainly through enhanced social connectedness and life satisfaction [35]. US studies similarly suggested that digital technology improved health indirectly via enriched social interaction, greater information access, and increased cognitive stimulation. Moreover, internet use benefited older adults’ mental health, with social capital partially mediating the effect [36]. In China, a persistent digital divide, especially in rural and low-education groups, further dampened any direct health effect [37].

What was notable in this study was the negative direct path from digital inclusion to overall health. This may reflect information overload, increased exposure to health misinformation [16], health perfectionism, driven social comparison, and health anxiety, all of which may exacerbate anxiety and offset positive spillovers transmitted through social engagement and mental health [25]. These findings emphasize that digitalization is not inherently health-promoting, while containing both enabling and burdensome properties. Policy should therefore prioritize not only access but also content quality, digital safety, and age-friendly platform design, embedding digital literacy within broader behavioral and psychosocial intervention strategies to unlock the genuine health-promoting potential of digital inclusion.

Third, the study verified that social engagement mediated between digital inclusion and perceived health, while mental health was the core bridging mechanism. This underscores the pivotal role of mental health in shaping health perceptions. The mechanism suggested is that positive experiences derived from social support, activity participation, and social interaction are re-processed psychologically—via reduced depressive symptoms, greater social adaptation, and higher life satisfaction—before they translate into better SRH. Thus, social engagement serves as an upstream facilitator of psychological well-being, whereas mental health functions as the final common pathway through which digital inclusion affects overall health.

This mechanism accorded with the WHO Active Ageing chain of “social connectedness → mental health → overall health” [38] and was supported by diverse empirical evidence. For example, the longitudinal Chinese Longitudinal Healthy Longevity Survey showed that the lack of participation predicted declines in mental health, whereas active participation was linked to better psychological well-being [10]; leisure-oriented participation could improve mental health broadly and significantly [39]. Additionally, the Australian Longitudinal Study on Women’s Health found that persistent or rising loneliness was closely tied to deterioration in mental health–related quality of life, highlighting the long-term protective value of social ties [40]. Korean aging panel studies showed that multimorbidity and disability depressed SRH, whereas social engagement partially mitigated these effects through psychological pathways [41]. In rural China, research on “empty-nest” older adults indicated that internet use directly raised life satisfaction and indirectly improved mental health by boosting participation, thereby further elevating health perceptions [42]. Moreover, a recent cross-national systematic review of digital interventions (randomized controlled trials) showed that online socialization and digital interaction significantly reduced loneliness and depressive symptoms, indirectly enhancing subjective health and life satisfaction [25]. These findings indicated that the dual mediation of social engagement and mental health is the key mechanism through which digital inclusion delivers health benefits.

Although the SEM results revealed significant links among digital inclusion, social engagement, mental health, and SRH, these pathways should not be interpreted as causal. The cross-sectional design prevents verification of temporal ordering, and reverse or bidirectional influences remain possible—for example, older adults with better health or stronger social ties may be more inclined to adopt digital technologies. Unobserved factors, such as cognitive status or personality traits, may also contribute to the associations observed. Thus, the mediation pathways identified here reflect theoretical plausibility rather than confirmed causal processes. Longitudinal analyses using future waves of CLASS will be necessary to assess whether these mechanisms hold over time.

Finally, the study revealed cross-national differences. Compared with high-income Western settings, the health benefits of digital inclusion among Chinese older adults relied more on indirect pathways (via social engagement and mental health) than on direct effects. Variations in social structure, welfare systems, and digital health ecosystem maturity likely contribute to these differences. In Europe and North America, digital tools were more deeply embedded in daily life and were closely integrated with community health services, social welfare, and family care, forming a “digital + in-person” support network that yielded more immediate perceived health gains [27]. In China, limited penetration of digital health services, uneven distribution of community digital resources, and the predominance of offline group activities among older adults meant that participation—although beneficial for mental health—had weaker direct links to concrete health behaviors (eg, screening, exercise, and self-management) [43]. Gaps also remained in the availability and quality of mental health services. Although mental health sat at the center of the causal chain, under-identification of emotional disorders in community settings and limited access to age-appropriate professional interventions likely attenuated its potential health benefits. By contrast, many high-income countries have embedded mental health supports within primary care and community systems, amplifying the health gains from both participation and digital technology.

### Limitations

This study had several limitations. First, SRH was a subjective measure without objective clinical or biomarker data. As it was susceptible to cognitive frames and momentary affect, potentially biasing associations and attenuating the precision of mediation estimates among digital inclusion, social engagement, and mental health. Second, the negative direct effect of digital inclusion on SRH may partly reflect perceptual or cognitive mechanisms—such as upward social comparison, exposure to idealized health information online, or heightened health anxiety—rather than a true adverse effect of digital engagement. This analysis was unable to fully disentangle these psychological influences from structural digital factors. Third, this study relied on cross-sectional data; thus, the directionality of associations cannot be conclusively established. The mediation effects suggested by SEM may partially reflect reverse causality or unmeasured confounding. Future research using multiwave CLASS data will allow for longitudinal validation of these mechanisms and more rigorous evaluations of causal ordering.

### Implications

On the basis of these findings, several implications emerge for policy, practice, and the design of future interventions. First, digital inclusion initiatives must extend beyond access provision to emphasize skills, usability, and sustained engagement, particularly for rural, low-education, and less digitally confident older adults. Integrating digital literacy with health education and psychosocial support may help ensure that digital engagement translates into meaningful health benefits rather than information overload or anxiety. Second, expanding opportunities for diverse and age-appropriate social participation—both online and offline—is essential, as participation serves as an upstream determinant of psychological well-being and a conduit through which digital inclusion exerts its influence on health. Community organizations, social service agencies, and primary care institutions should co-develop engagement platforms that are socially meaningful, culturally appropriate, and accessible to older adults with varying functional capacities.

### Conclusions

This study shows that digital inclusion shapes older adults’ health perceptions primarily through indirect pathways, operating first by enhancing social engagement and subsequently by improving mental health. While demographic and health-related vulnerabilities such as aging, chronic diseases, and functional limitations remain dominant predictors of poorer SRH, digital competence and meaningful participation represent actionable levers that can mitigate health disadvantages in later life. Mental health emerged as the pivotal link in the causal chain, underscoring the need to position psychological well-being at the center of healthy aging strategies. These findings highlight the importance of moving beyond infrastructure-focused digital policies to approaches that build skills, support sustained social participation, and integrate mental health services within both community and digital ecosystems. As population aging and digital transformation continue to accelerate, coordinated interventions that align technological, social, and psychological resources will be essential for promoting active, equitable, and healthy aging.

## Abbreviations

ADL: basic activities of daily living
CLASS: China Longitudinal Aging Social Survey
ELSA: English Longitudinal Study of Ageing
HRS: Health and Retirement Study
SEM: structural equation modeling
SHARE: Survey of Health, Ageing and Retirement in Europe
SRH: self-rated health
WHO: World Health Organization
